# Pre-clinical and clinical studies on the role of RBM3 in muscle-invasive bladder cancer: longitudinal expression, transcriptome-level effects and modulation of chemosensitivity

**DOI:** 10.1186/s12885-021-09168-7

**Published:** 2022-02-02

**Authors:** Sara Wahlin, Karolina Boman, Bruce Moran, Björn Nodin, William M. Gallagher, Emelie Karnevi, Karin Jirström

**Affiliations:** 1 Division of Oncology and Therapeutic Pathology, Department of Clinical Sciences, Lund University, Lund, Sweden; 2 UCD School of Biomolecular and Biomedical Sciences, UCD Conway Institute, University College Dublin, Belfield, Dublin, Ireland

**Keywords:** RBM3, Biomarker, Cell cycle, Prediction, Chemotherapy response, Muscle-invasive bladder cancer

## Abstract

**Background:**

The response to neoadjuvant cisplatin-based chemotherapy (NAC) in muscle-invasive bladder cancer (MIBC) is impaired in up to 50% of patients due to chemoresistance, with no predictive biomarkers in clinical use. The proto-oncogene RNA-binding motif protein 3 (RBM3) has emerged as a putative modulator of chemotherapy response in several solid tumours but has a hitherto unrecognized role in MIBC.

**Methods:**

RBM3 protein expression level in tumour cells was assessed via immunohistochemistry in paired transurethral resection of the bladder (TURB) specimens, cystectomy specimens and lymph node metastases from a consecutive cohort of 145 patients, 65 of whom were treated with NAC. Kaplan-Meier and Cox regression analyses were applied to estimate the impact of RBM3 expression on time to recurrence (TTR), cancer-specific survival (CSS), and overall survival (OS) in strata according to NAC treatment. The effect of siRNA-mediated silencing of RBM3 on chemosensitivity was examined in RT4 and T24 human bladder carcinoma cells in vitro. Cellular functions of RBM3 were assessed using RNA-sequencing and gene ontology analysis, followed by investigation of cell cycle distribution using flow cytometry.

**Results:**

RBM3 protein expression was significantly higher in TURB compared to cystectomy specimens but showed consistency between primary tumours and lymph node metastases. Patients with high-tumour specific RBM3 expression treated with NAC had a significantly reduced risk of recurrence and a prolonged CSS and OS compared to NAC-untreated patients. In high-grade T24 carcinoma cells, which expressed higher *RBM3* mRNA levels compared to RT4 cells, RBM3 silencing conferred a decreased sensitivity to cisplatin and gemcitabine. Transcriptomic analysis revealed potential involvement of RBM3 in facilitating cell cycle progression, in particular G_1_/S-phase transition, and initiation of DNA replication. Furthermore, siRBM3-transfected T24 cells displayed an accumulation of cells residing in the G_1_-phase as well as altered levels of recognised regulators of G_1_-phase progression, including Cyclin D1/CDK4 and CDK2.

**Conclusions:**

The presented data highlight the potential value of RBM3 as a predictive biomarker of chemotherapy response in MIBC, which could, if prospectively validated, improve treatment stratification of patients with this aggressive disease.

**Supplementary Information:**

The online version contains supplementary material available at 10.1186/s12885-021-09168-7.

## Background

The quest for molecular determinants that could advance our understanding of the biological behaviour of tumour cells, and add prognostic and predictive guidance for refining treatment strategies, has resulted in the characterization of several promising candidates, including RNA-binding motif protein 3 (RBM3).

RBM3, originally discovered as a cold-shock protein [1], has pleiotropic cellular functions. With its DNA and RNA binding capabilities [2], RBM3 promotes global protein synthesis [3], the stability of mRNA bearing AU-rich elements [4], and posttranscriptional biogenesis of microRNAs [5], thus exerting broad regulatory influences on the proteome [6]. RBM3 is induced in response to cellular stress, e.g. endoplasmatic reticulum (ER) stress, hypothermia and hypoxia, to mediate cell protection by attenuating both apoptosis and necrosis [1, 7, 8]. This causality has been illuminated within the research context of brain ischemia, where RBM3 has demonstrated an indispensable role in the neuroprotective effects of therapeutic hypothermia after hypoxic ischemia [9]. In addition, RBM3 is described as a proto-oncogene, promoting cell cycle progression and preventing mitotic catastrophe [4]. The RBM3 expression status has been highlighted as a potentially useful biomarker for prognostication and treatment responsiveness in multiple malignancies. High RBM3 expression has been shown to signify an improved prognosis in solid cancers including malignant melanoma [10], colorectal [11, 12], urothelial bladder [13, 14], breast [15], and epithelial ovarian cancer [16] (reviewed in [17]). Contrastingly, in pancreatic cancer, high RBM3 levels correlated to reduced survival [18]. Moreover, in vitro studies have reported decreased sensitivity to chemotherapy after RBM3 suppression in epithelial ovarian and pancreatic cancer cells [16, 18].

While upregulation of RBM3 expression in urothelial bladder cancer has been identified as an independent factor of a favourable outcome in studies encompassing tumours of all clinical stages, its prognostic and in particular predictive value in muscle-invasive bladder cancer (MIBC) remains unclear. In MIBC, such biomarkers would be of indisputable importance as the survival benefit of standard treatment with neoadjuvant cisplatin-based chemotherapy (NAC) prior to radical cystectomy is limited to 30–50% of patients due to chemoresistance [19]. Importantly, NAC treatment has a substantial impact on survival in responding patients, especially in complete responders (i.e. pT0N0), whereas non-responding patients are at risk of severe toxicity and surgical delay [19, 20]. Analysis of the highly heterogeneous genomic landscape of MIBC in the context of chemosensitivity have identified several tumour characteristics that may serve as predictive markers of therapeutic efficacy. Somatic mutations in DNA repair-associated genes, including *ATM, RB1* and *FANCC* [21], and *ERBB2* [22] have been associated with response to cisplatin-based chemotherapy. *ERCC2* mutations have been shown to be sufficient to drive cisplatin-sensitivity in xenograft models [23] and to correlate with NAC response [24], however not in all studies [22]. Taber et al. recently demonstrated a link between genomic instability driven by chromosomal alterations, indels and *BRCA2* mutations and improved response rates, in addition to immune cell infiltration and PD-1 protein expression [25]. Furthermore, molecular subtype-based analyses have yielded contrasting results [26], where basal tumours have been associated with an increased overall survival following NAC treatment [27], while enrichment of non-responders within the basal/squamous subtype has been reported [25]. However, as no robust predictive biomarkers have yet been implemented in clinical use, further profiling of pre-treatment transurethral resection of the bladder (TURB) specimens is needed in order to provide decisive insights into the mechanisms underlying chemotherapy response and identify novel biomarkers that could aid treatment selection [28].

The aim of this study was therefore to examine the putative role of RBM3 as a prognostic and predictive biomarker in relation to NAC in MIBC. To this end, RBM3 protein expression was examined by immunohistochemistry (IHC) in paired primary tumour samples from TURB and cystectomy specimens, respectively, as well as a subset of synchronous lymph node metastases from a consecutive cohort of 145 patients. Furthermore, the potentially modifying effect of RBM3 suppression on chemosensitivity was assessed in vitro*,* and functional genomics was applied to delineate biological processes associated with RBM3.

## Methods

### Study cohort

A previously detailed [29] retrospective consecutive series of 145 patients diagnosed with MIBC having undergone TURB and ensuing cystectomy at Skåne University Hospital, Malmö, Sweden, between January 1st 2011 and December 31st 2014, was included in the present study. Paired tissue specimens from TURB (*n* = 145), cystectomy (*n* = 135) and lymph node metastases (*n* = 27) could be retrieved. All cases were histopathologically re-evaluated by a board-certified pathologist (KJ). Clinical information was obtained from medical records. Follow-up started at MIBC diagnosis and ended at death or August 31st 2018. One hundred and fifteen (79.3%) patients had been diagnosed with de novo MIBC. Prior Bacillus Calmette-Guérin (BCG) treatment was denoted in 13 (9.0%) patients, NAC treatment with methotrexate, vinblastine, adriamycin and cisplatin (MVAC) in 65 (44.8%) patients and adjuvant chemotherapy in 12 (8.3%) patients. Treatment response was based on pathological evaluation of tissue specimens from radical cystectomy. Complete response (pT0N0) was denoted in 26/65 (40.0%) and 6/80 (7.5%) patients treated with radical cystectomy with and without prior NAC treatment, respectively. Approval for the study was obtained from the Ethics committee at Lund University (reference number 445-2007), whereby the committee waived the need for informed consent other than the option to opt out. All methods were carried out in accordance with relevant guidelines and regulations.

### Tissue microarray construction and immunohistochemistry

Tissue microarrays (TMAs) were constructed with triplicate 1 mm cores from each of the different tissue specimens, i.e. TURB specimens, cystectomy specimens and lymph node metastases, using a semi-automated arraying device (TMArrayer, Pathology Devices, Westminster, MD, USA). All core biopsies were taken from representative tumour areas and when possible from different donor paraffin blocks. Four μm TMA-sections were automatically pretreated with the PT Link system (Dako, Copenhagen, Denmark) with target retrieval solution buffer pH 6, and immunohistochemically stained in an Autostainer Plus (Dako) with the human monoclonal anti-RBM3 antibody (AMAb90655, RRID:AB_2665621, dilution 1:750, Atlas Antibodies AB, Stockholm, Sweden). The specificity of the antibody has been previously validated [16]. RBM3 staining was annotated by two independent observers (SW and KJ) blinded to clinical data. Cases with missing TMA cores or cores with an insufficient amount of tumour cells, in addition to cystectomy specimens from cases with pT0 (*n* = 35), were excluded from the subsequent analyses. RBM3 was predominantly expressed in the tumour cell nuclei, whereby the fraction of nuclear positivity (NF) was categorized as 0 (0–1%), 1 (2–25%), 2 (26–50%), 3(51–75%) and 4 (> 75%), and the intensity (NI) as 0 (negative), 1 (weak), 2 (moderate) and 3 (strong). In cases with heterogeneous RBM3 intensity, the dominating staining pattern was denoted. A combined nuclear score (NS) was constructed, i.e. a multiplier of NF and NI. As cut off values for dichotomization of RBM3 expression into high versus low could not be established by Classification and regression tree (CRT) analysis, the median value of the NS for each tissue specimen was used for subsequent analyses. IHC images were captured using the VS120 Olympus with OlyVIA software v3.2 (Olympus Corporation, Tokyo, Japan).

### Cell culture

Human bladder cancer cell lines RT4 (grade 1, RRID:CVCL_0036) and T24 (grade 3, RRID:CVCL_0554) were purchased from Sigma-Aldrich (St Louis, MO, USA). The cells were cultured in McCoy’s 5a medium supplemented with 10% fetal bovine serum (FBS), 1% L-glutamine, 100 U/mL penicillin and 100 μg/mL streptomycin in a humified 5% CO_2_ atmosphere at 37 °C. All reagents for the in vitro experiments were purchased from ThermoFisher Scientific (Waltham, USA) unless stated otherwise.

### siRNA transfection

siRNA transfection was performed in a similar manner as previously described [18]. Bladder cancer cells were seeded in T-25 flasks (5 × 10^5^ cells) and incubated for 24 h at 37 °C. Next, cells were washed twice in phosphate buffered saline (PBS) and resuspended in growth medium without FBS. Cells were transfected with non-targeting negative siRNA control (Silencer™ Select Negative control No.1 siRNA, catalog number 4390843) or anti-RBM3 (s11858 + s11860) siRNA using Lipofectamine 2000, diluted in OptiMEM to a final siRNA concentration of 25 nM. After 4.5 h the transfection was stopped, the medium changed to full growth medium and the cells were left to recover overnight. The following day, cells were harvested and spun down to pellets. The pellets were either fixated, dehydrated and embedded in paraffin for immunocytochemistry or resuspended in TRIzol and stored at − 20 °C for qPCR.

### Immunocytochemistry

TMAs were constructed from paraffin-embedded cell pellets of RT4 and T24 cells and immunohistochemically stained according to the same protocol as for the formalin-fixed paraffin-embedded tissue specimens. Representative images were taken using cellSens Dimension software (Olympus) at 20X magnification.

### Quantitative PCR (qPCR)

The cell samples were thawed and RNA purification was performed using TRIzol with phasemaker tubes according to manufacturer’s instructions. RNA cleanup was performed using RNeasy minelute kit (QIAGEN) and the RNA concentration was determined using Qubit with the RNA HS kit. Prior to qRT-PCR, cDNA reverse transcription was performed with the High-capacity cDNA reverse transcription kit and total cDNA concentration was determined using Qubit with the DNA HS kit. Ten ng per reaction of each sample was used for subsequent qRT-PCR with RBM3, CCND1, CCND3, CCNG1, CDK2, CDK4, and CDKN1B TaqMan gene expression assay (Assay ID Hs00943160_g1, Hs00765553_m1, Hs05046059_s1, Hs00171112_m1, Hs01548894_m1, Hs00364847_m1 and Hs00153277_m1, respectively), with samples run in triplicates. 18S served as endogenous control (Assay ID Hs039288985_g1).

### Cell viability assay

Following siRNA transfection and 24 h incubation with regular growth medium, cells were harvested and reseeded in 96-well plates (2 × 10^4^ cells per well). The following day, cells were subjected to cisplatin (0–250 μM) or gemcitabine (0–250 nM) for 24 or 30 h, respectively, in regular growth medium. WST-1 was added to the wells and the plates were read at 450 nm after 1 h, with a reference wavelength of 620 nm. Cell viability of non-chemotherapy treated siRBM3-transfected and non-targeting siRNA control cells was measured at 24, 30 and 72 h.

### Cell cycle analysis

Cells were plated in 6-well plates (1-2 × 10^5^) and incubated for 72 h at 37 °C. The cells were transfected with siRNA against RBM3 or non-targeting negative control for 4.5 h. The following day, cells were harvested by trypsinization, counted, washed with PBS and fixated (1 × 10^6^ per sample) in ice cold 70% ethanol. The samples were stored at -20 °C until flow cytometry. Prior to cell cycle analysis, cells were washed with PBS and resuspended in 500 μL Propidium Iodide (PI) solution (Sigma-Aldrich). Samples were run using BD Accuri C6 (BD Biosciences, Mississauga, Canada) and 2 × 10^4^ events were collected of each sample. The cell populations were gated and subjected to doublet discrimination to identify single cells, followed by application of the Watson Pragmatic algorithm for gating of G0/G1, S and G2/M cell populations using FlowJo software v10.6.1.

### Western immunoblotting

Cells were seeded in 6-well plates (2 × 10^5^ cells per well) and incubated for 48 h at 37 °C prior to siRNA-mediated RBM3 silencing. The following day, cells were washed with PBS, lysed on ice for 10 min in lysis buffer (10 mM Tris-HCl, 50 mM NaCl, 5 mM EDTA, 30 mM sodium pyrophosphate, 50 nM sodium fluoride, 100 μM sodium orthovanadate, 1% Triton X100, pH 7.6) and stored at -20 °C. Protein quantification was performed using Pierce BCA Protein Assay Kit according to manufacturer’s instructions and 20 μg was used from each sample. The samples were denatured in Laemmli sample buffer (Sigma-Aldrich), boiled for 5 min at 95 °C and placed on an 8–16% gradient gel (Bio-rad Laboratories, Hercules, USA) with high range rainbow markers at both ends (GE Healthcare Life Sciences). Following electrophoresis, wet tank transfer was performed, and proteins were transferred to a 0.45 μm nitrocellulose membrane and dried for 1 h. Total protein staining was performed using Revert 700 (LI-COR Biosciences, Lincoln, USA), imaged at 700 nm. The membrane was destained and blocked with Intercept TBS blocking buffer (LI-COR). Following blocking, the membrane was cut and primary antibody incubation was performed overnight at 4 °C with anti-GAPDH (Millipore 1:1000) or anti-RBM3 (AMAb90655, 1:1000). The membrane was subsequently washed and incubated with secondary IRDye 800CW goat anti-mouse (LI-COR) for 1 h at room temperature (GAPDH 1:15000, RBM3 1:5000). The secondary antibody was thoroughly rinsed off, followed by near-infrared (NIR) protein detection using a LI-COR Biosciences Odyssey Imaging System. Images were analysed using Image studio software (LI-COR). Protein quantification was performed in Empiria Studio Software (LI-COR) by normalizing each lane against total protein content and the relative protein concentration after siRNA transfection compared to control was calculated.

### RNA-sequencing

T24 cells were transfected with anti-RBM3 siRNA or non-targeting siRNA control, as described above. RNA purification was performed according to the qPCR protocol and samples were prepared in triplicate. RNA quantification and quality assessment were performed using Nanodrop 1000 (Mason Technology, Dublin, Ireland) and Bioanalyzer 2100 (Agilent, Santa Clara, USA). cDNA libraries were prepared from the RNA samples using TruSeq Stranded mRNA Library Prep Kit on the NeoPrep instrument (Illumina, San Diego, USA) according to manufacturer’s instructions, and sequenced (single end 1 × 75 bp) using the NextSeq 500 platform (Illumina). Fastq files were downloaded from the Illumina BaseSpace using the BaseSpace download tool and the quality of the files was determined using FastQC. Data were trimmed of sequencing adaptors and low-quality base calls using BBDuk tool in the BBMap package. Alignment to the human hg19/GRCh37 genome reference was done using STAR version 2.5.2a [30]. Duplicate reads were marked using Picard MarkDuplicates. Read counts were produced by the featureCounts tool from the SubRead package [31], combined for all samples and used as input for analysis of differential gene expression. Differential expression gene (DEG) analysis was conducted using the R package DESeq2 [32]. Gene ontology (GO) enrichment analysis for detection of altered cellular pathways were applied using the Gene Ontology enrichment analysis and visualization tool (GOrilla) [33]. DEGs with fold change ±1.5 and false discovery rate (FDR) < 0.01 were used as input for enrichment analysis. GO terms with Benjamini-Hochberg multiple testing corrected FDR *q*-value < 0.05 were considered significantly enriched.

### Statistical analysis

Wilcoxon signed-rank test was used for comparison of biomarker expression in paired tissue specimens. Chi-square test and Fisher’s Exact test for categorical variables and Mann-Whitney U test for continuous variables were applied to examine associations between RBM3 expression and clinicopathological characteristics. *P*-values were adjusted for multiple testing using the Holm-Bonferroni method. Kaplan-Meier estimates and log-rank tests were used to examine differences in overall survival (OS), cancer-specific survival (CSS) and time to recurrence (TTR) in combined strata according to RBM3 expression and NAC treatment. TTR was defined as time from TURB to the date of recurrent disease or death from bladder cancer. Cox regression proportional hazard models were used to estimate hazard ratios (HRs) for the impact of RBM3 levels on OS, CSS, and TTR in univariable and multivariable analysis, adjusted for age at diagnosis, pathological tumour stage at cystectomy, nodal stage, neoadjuvant, and adjuvant chemotherapy. For assessment of a potential treatment interaction between RBM3 and NAC, an interaction variable was constructed of NAC status (±) x dichotomous RBM3 expression (low/high). The interaction term was analysed in relation to OS, CSS and TTR using Cox regression analysis, where the univariable model included NAC status, the binary covariate of RBM3 expression and the interaction variable, and the multivariable model was adjusted for the above-mentioned parameters. For in vitro experiments, unpaired *t* test and non-linear regression were used. Data are presented as mean ± SEM derived from at least three independent experiments. Statistical analyses were performed using IBM SPSS Statistics version 25 (SPSS Inc., Chicago, IL, USA) for clinical data, GraphPad Prism version 9 (GraphPad Software, LA Jolla, CA, USA) for experimental data and RStudio Version 1.2.5033 (RStudio Team, Boston, MA, USA) for sequencing data. Graphs were constructed using GraphPad. All statistical tests were two-sided and *p*-values < 0.05 were considered significant.

## Results

### Longitudinal nuclear RBM3 expression in paired tissue specimens

**Fig. 1:**
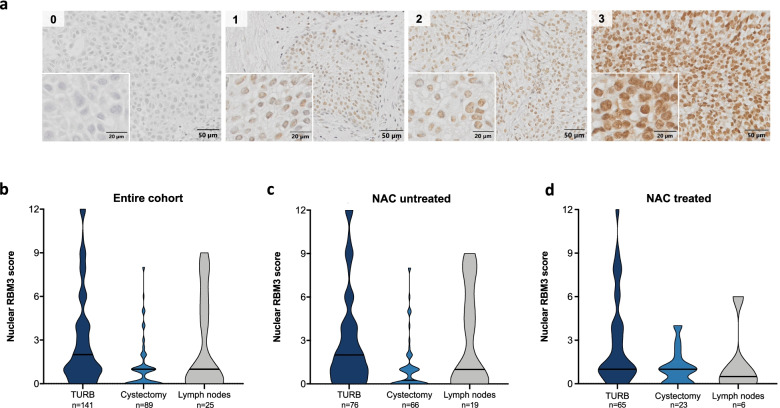
RBM3 expression in muscle-invasive bladder cancer. **a** Representative immunohistochemical images of nuclear RBM3 expression with staining intensity denoted as negative (0), weak (1), moderate (2) and strong (3). Scale bars represent 50 μm (10x) with 20 μm (40x) insertion. Violin plots of the distribution of nuclear RBM3 expression (multiplier of fraction and intensity, range 0–12) across tissue specimens from **b** entire cohort, **c** NAC-untreated cases and **d** NAC-treated cases. Median values are presented (black lines). TURB, transurethral resection of the bladder

Tumour-specific RBM3 protein expression could be evaluated in TURB specimens from 141/145 (97.2%) cases, in cystectomy specimens from 89/135 (65.9%) cases and in lymph node metastases from 25/27 (92.6%) cases. Representative images of RBM3 immunostaining and the distribution of RBM3 expression across tissue samples are shown in Fig. 1a-d. Analysis of RBM3 expression in paired tissue samples was performed using Wilcoxon-signed rank test. For the entire cohort, significantly higher RBM3 expression levels were denoted in TURB specimens compared to cystectomy specimens (*p* < 0.001). There were no significant differences in RBM3 expression between primary tumours and lymph node metastases, neither for TURB nor cystectomy specimens (*p* = 0.548 and *p* = 0.344, respectively). After stratification according to NAC treatment, the difference in RBM3 expression between TURB and cystectomy specimens remained significant in NAC-untreated patients (*p* < 0.001), and a similar trend was also indicated in NAC-treated patients (*p* = 0.053) (Additional file 1: Fig. S1a, b).

For subsequent statistical analyses, RBM3 expression was categorized into low versus high expression based on median values of the nuclear score across TMA cores for each case and specimen type (see Fig. 1b). For TURB specimens, the median value was 2.0, rendering 57/141 (40.4%) cases with high expression, and for cystectomy specimens the median value was 1.0, rendering 20/89 (22.5%) cases with high expression. Notably, while the RBM3 expression was significantly higher in TURB specimens compared to cystectomy specimens, a shift from low RBM3 expression in TURB specimens to high RBM3 expression in cystectomy specimens was recorded in ten out of 89 (11.2%) cases.

### Associations of RBM3 expression with clinicopathological characteristics

The distribution of patient and tumour characteristics of the study cohort according to RBM3 expression is presented in Additional file 2: Table S1. A sub-analysis of patients from whom paired TURB specimens and cystectomy specimens could be assessed are demonstrated in Additional file 3: Table S2. No significant correlations between biomarker expression and established clinicopathological factors were observed.

### Associations of RBM3 expression with histopathological response

**Fig. 2:**
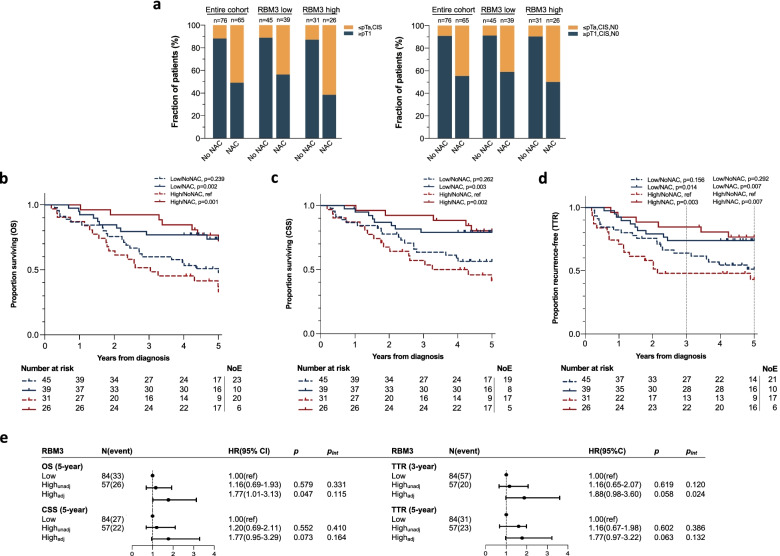
Histopathological response and risk of recurrence according to neoadjuvant chemotherapy and RBM3 expression. **a** The proportion of patients with pathological downstaging of the primary tumour (pT0, Ta, CIS) as well as pathological non-invasive downstaging (≤pTa, CIS, N0) in the entire cohort and according to RBM3 expression. Kaplan-Meier estimates of **b** 5-year overall survival (OS), **c** 5-year cancer-specific survival (CSS) and **d** 3- and 5-year time to recurrence (TTR) after diagnosis stratified according to dichotomous RBM3 expression in TURB specimens and neoadjuvant chemotherapy. *P*-values are derived from log-rank test for pairwise comparison, with high RBM3/No NAC as the reference group (ref). NoE, number of events. **e** Forest plot illustrating hazard ratio with 95% confidence interval (CI) and *p*-values (*p*) from uni- and multivariable Cox proportional hazards analysis of 5-year OS, 5-year CSS and 3- and 5-year TTR, respectively. Multivariable model adjusted for age at diagnosis (continuous), T-stage at cystectomy, N-stage, neoadjuvant, and adjuvant chemotherapy. *p*_int_: *p*-value for interaction derived from univariable and multivariable Cox regression analysis of OS, CSS and TTR, respectively, which included a term of interaction by multiplication of NAC status (±) and the binary covariate of RBM3 expression (low/high)

The correlation between RBM3 expression in TURB specimens and histopathological response to NAC treatment was next evaluated (Fig. 2a). In the entire cohort, downstaging of the primary tumour to ≤pTa/CIS was observed in 33/65 (50.8%) of the NAC-treated patients, out of whom 29/65 (44.6%) experienced pathological non-invasive downstaging to ≤pTa, CIS, N0. Further analysis of NAC-treated patients according to RBM3 expression showed that the fraction of pathological downstaging of the primary tumour was higher in patients with high RBM3 expression compared to low RBM3 expression; however, this was not statistically significant (*p* = 0.156). A similar, although less evident, trend was also seen for pathological non-invasive downstaging to ≤pTa, CIS, N0 following NAC.

### Prognostic and predictive significance of RBM3 expression

To assess the potential prognostic and predictive value of RBM3 expression, Kaplan-Meier analyses of OS, CSS and TTR were conducted in combined strata according to biomarker expression in TURB specimens and NAC treatment. At 5-year follow up, 59/141(41.8%) patients had died, 49/59(83.1%) of whom due to MIBC, and 54/141(38.3%) had denoted recurrent disease. As shown in Fig. 2b, c, NAC-untreated patients with high RBM3 tumoural expression had a significantly reduced OS and CSS compared to NAC-treated patients (*p* = 0.001 and *p* = 0.002, respectively). RBM3 expression was not prognostic in relation to OS and CSS in univariable Cox regression analysis (Fig. 2e). In multivariable analysis, adjusted for age at diagnosis, T-stage at cystectomy, N-stage, NAC, and adjuvant chemotherapy that have previously been shown to be prognostic factors for the herein investigated cohort [29], high RBM3 expression was found to be independently associated with an impaired OS (HR = 1.77; 95% CI 1.01–3.13). A similar, however non-significant, trend was observed for CSS (HR = 1.77; 95% CI 0.95–3.29).

Since most local recurrences manifest during the first 24 months and distant metastases within 3 years after radical cystectomy [34], analysis of TTR at both 3- and 5-year follow-up was performed. The lowest proportion of recurrence-free patients was observed for NAC-untreated patients with high tumoural RBM3 expression, which served as the reference group for pairwise comparison between the investigated strata (Fig. 2d). Interestingly, patients with high tumoural RBM3 expression not receiving NAC had a significantly higher proportion of recurrences compared to NAC-treated patients (*p* = 0.007), where the largest difference in risk of recurrence between these patient groups was observed during the first 3 years after diagnosis (*p* = 0.003). In univariable Cox regression analysis of the risk of recurrence of MIBC within 3 and 5 years, respectively, RBM3 expression was not prognostic (Fig. 2e). In multivariable analysis, a trend, however non-significant, towards a higher risk of recurrence was denoted in patients with high RBM3 expression (HR = 1.88; 95% CI 0.98–3.60 and HR = 1.77; 95% CI 0.97–3.22 for 3 and 5 years, respectively). A potential treatment interaction between NAC and RBM3 expression was assessed by inclusion of an interaction term, i.e. multiplier of NAC status (yes/no) and dichotomous RBM3 expression (low/high), to the univariable and multivariable Cox regression models. No significant treatment interaction between NAC and RBM3 expression could be seen in relation to OS and CSS. However, in relation to TTR, a significant treatment interaction (*p* = 0.024) between NAC and RBM3 expression was observed in the adjusted model during the first 3 years after diagnosis, but did not remain significant in the analysis based on 5-year follow-up (Fig. 2e).

### RBM3 suppression impairs sensitivity to chemotherapy in vitro

**Fig. 3:**
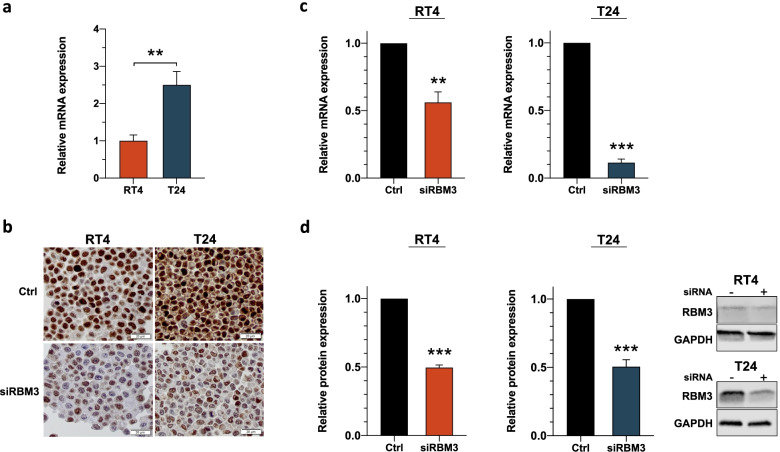
Decreased RBM3 levels after siRNA transfection. **a** qRT-PCR analysis of relative *RBM3* mRNA expression levels in RT4 and T24 cells normalized to control (18S). **b** Visualization of RBM3 protein expression in bladder cancer cell lines RT4 and T24 after siRNA-mediated RBM3 knockdown compared to non-targeting siRNA control. Representative images taken with cellSens Dimension software at 20X magnification. Scale bar represents 20 μm. **c** Reduced *RBM3* mRNA levels after siRNA transfection compared to non-targeting siRNA control measured by qRT-PCR. **d** RBM3 protein expression levels after siRNA transfection compared to non-targeting siRNA control detected via Western blot. Quantification of relative protein expression, normalized to total protein content, was performed using Empiria Studio Software of samples derived from the same experiment and with blots processed in parallel. Images shown represent one of three independent experiments and are cropped from full-length blots presented in Additional file 4: Fig. S2. Bars represent mean ± SEM from at least three independent experiments performed in triplicate. **p < 0.01, ***p < 0.001, two-tailed unpaired *t* test

Given that RBM3 expression was frequently denoted in the MIBC cohort (84.8% of patients) and the finding of a reduced risk of recurrence and a prolonged survival in NAC-treated patients with high RBM3 expression, we sought to elucidate mechanisms related to RBM3 function in MIBC using an in vitro model of the well-characterized human bladder cancer cell lines RT4 and T24, of which the latter represents invasive disease [35]. At baseline, T24 cells displayed 2.5-fold higher *RBM3* mRNA expression levels than RT4 cells (*p* = 0.005, Fig. 3a). The cells were transfected with siRNA targeting RBM3 and non-targeting negative siRNA control. Representative IHC images of siRBM3-transfected and control cells are displayed in Fig. 3b. Following transfection, qRT-PCR and Western blot analyses demonstrated significantly reduced mRNA and protein RBM3 levels (Fig. 3c-d, Original Western blots are presented in Additional file 3: S2).

**Fig. 4:**
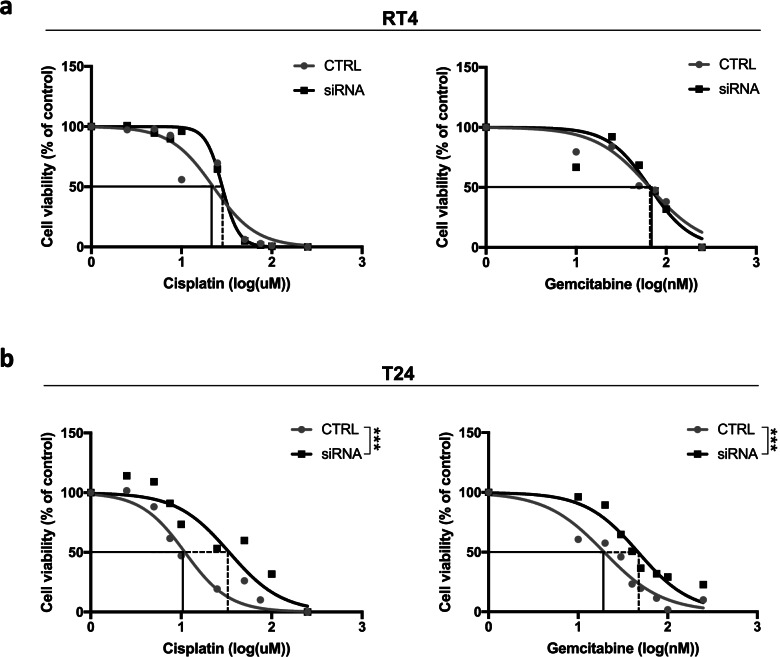
RBM3 knockdown increases cell viability upon chemotherapy treatment in vitro. Viability of **a** RT4 and **b** T24 RBM3 siRNA silenced (black lines) and non-targeting siRNA control (grey lines) treated bladder cancer cells after exposure to increased doses of cisplatin and gemcitabine. Cells were treated in quadruplicate wells for 24 h (cisplatin) or 30 h (gemcitabine) and the viability was measured using WST-1. Differences in IC_50_ values between siRNA and negative control were calculated by non-linear regression of normalized values, relative to control (untreated cells, 100% cell viability). The graph represents one of three independent experiments. ***p < 0.001

Next, the potential chemomodulating effect of RBM3 was addressed. Following transfection with siRBM3 and non-targeting negative siRNA control, RT4 and T24 cells were exposed to cisplatin or gemcitabine for 24 and 30 h, respectively, due to the kinetic differences of the two drugs. After 24 h of incubation with cisplatin, a minor shift towards reduced sensitivity to cisplatin treatment could be observed for RBM3 silenced RT4 cells compared to control. Suppression of RBM3 had no influence on the sensitivity to gemcitabine (Fig. 4a). In T24 cells, RBM3 silencing resulted in a significant 1.5-fold higher half-maximal inhibitory concentration of cisplatin compared to control (IC_50_ 1.53 log μM and 1.04 log μM, respectively, *p* < 0.001). Similarly, when T24 cells were exposed to gemcitabine, siRNA cells displayed a 1.3-fold significant increase in IC_50_ compared to control (1.70 log nM and 1.29 log nM, respectively, *p* < 0.001; Fig. 4b). In addition, when non-chemotherapy treated cells were compared to assess the influence on proliferation by silencing of RBM3 alone, no significant differences could be seen between non-targeting siRNA control and siRBM3 transfected RT4 and T24 cells, respectively (Additional file 5: Fig. S3a, b). Therefore, the observed changes in cell viability can be considered to be assigned to altered chemosensitivity after silencing of RBM3.

### Mapping of RBM3-associated cellular processes

**Fig. 5:**
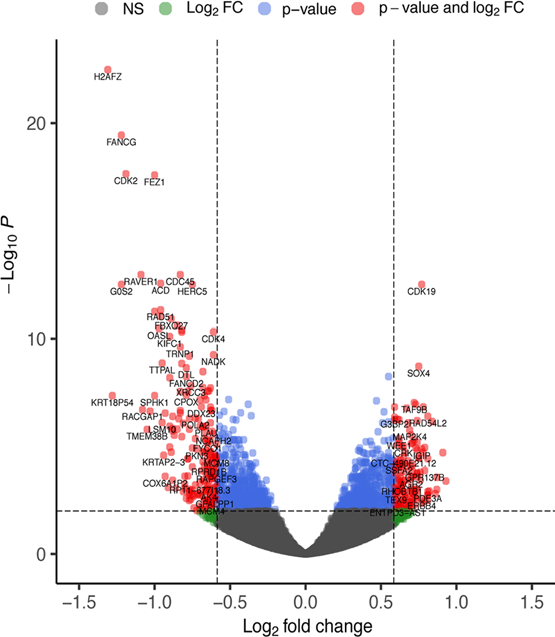
RBM3 related genes. Volcano plot illustrating differentially expressed genes based on RNA-sequencing of siRBM3-treated and non-targeting siRNA control T24 cells, showing 197 and 145 significantly up- and downregulated genes, respectively, with fold change of ≤1.5/≥ 1.5, and FDR < 0.01 (out of total 13,627 genes detected). NS, Not significant. FC, fold change

**Fig. 6:**
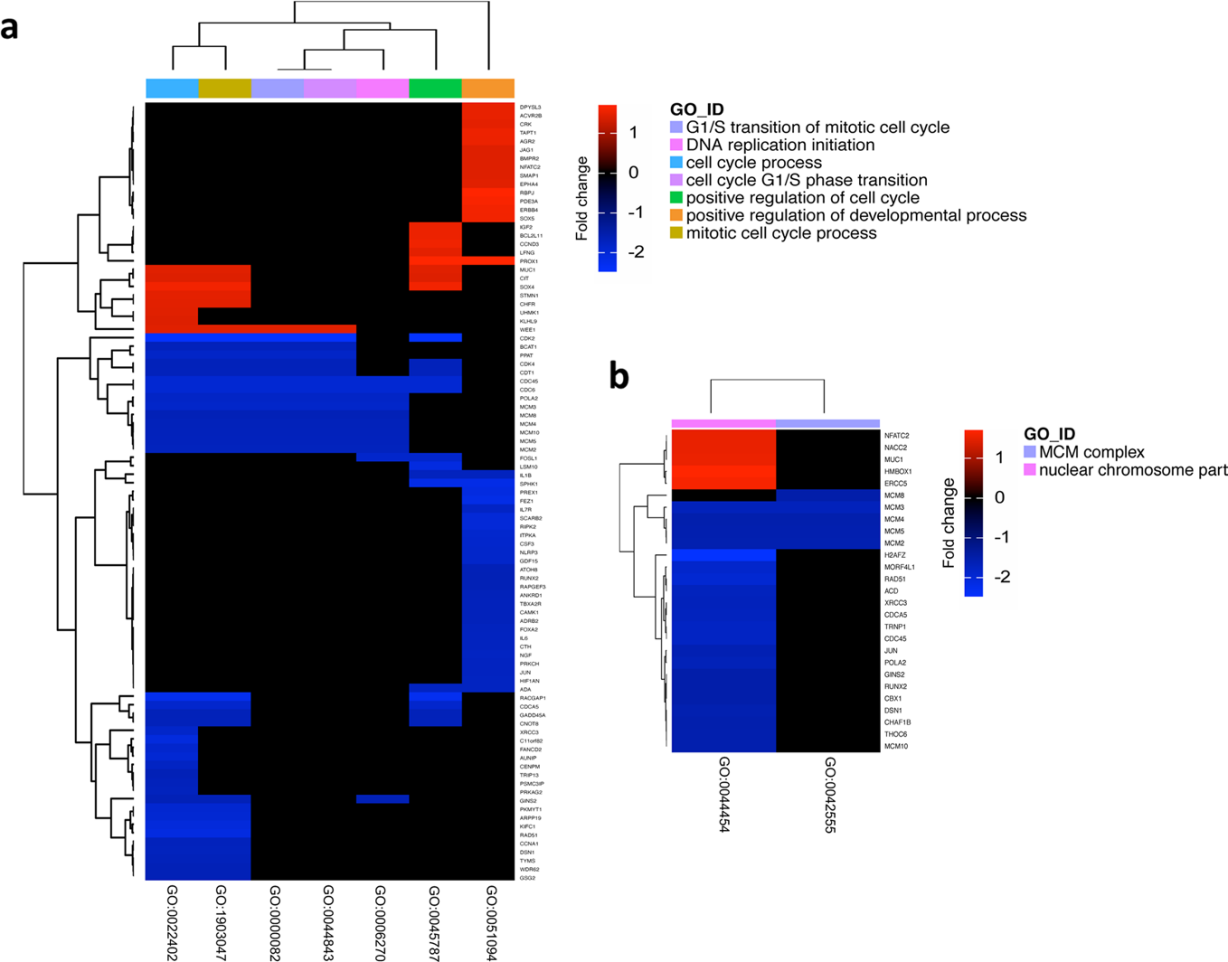
RBM3 associated biological processes. **a** Heat map representing biological processes derived from Gene Ontology enrichment analysis (GO terms, x-axis) of the significantly up- and downregulated differentially expressed genes (y-axis, FDR < 0.05). **b** Heat map of the cellular component derived from GO analysis (x-axis) of the significant differentially expressed genes (y-axis, FDR < 0.05). MCM, mini-chromosome maintenance protein complex

In an attempt to decipher the transcriptome level effects of RBM3, the gene expression profiles of siRBM3-treated and control T24 cells were subsequently analysed using RNA-sequencing. This resulted in a large number of DEGs (Fig. 5), where the significantly up- and downregulated DEGs (*n* = 197 and *n* = 145, respectively) were subjected to further GO analysis using GOrilla. Suppression of RBM3 resulted in significantly enriched GO terms for involvement of downregulated genes in a number of biological processes, including positive regulation of developmental processes, cell cycle processes, positive regulation of the cell cycle, G_1_/S-phase transition, and initiation of DNA replication. The upregulated genes were mainly involved in positive regulation of developmental processes, regulation of cell cycle and cell cycle processes (FDR < 0.05, Fig. 6a). In cellular component analysis, the significantly DEGs were found to be associated with GO terms for nuclear chromosome part and the mini-chromosome maintenance (MCM) protein complex (FDR < 0.05, Fig. 6).

### RBM3 influences G_1_/S-transition

**Fig. 7:**
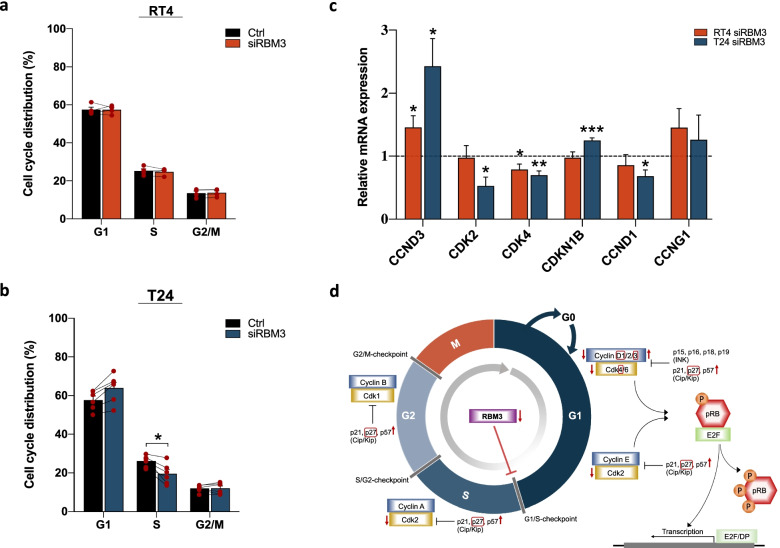
RBM3 silencing halts cell cycle progression in T24 bladder cancer cells. Following collection of data for 2 × 10^4^ cells, the cell population was gated and doublet discrimination was performed to identify single cells. Cell cycle analysis with the Watson Pragmatic algorithm was applied using FLowJo v10.6.1. on the single cell population for each sample. **a** In RT4 bladder cancer cells, no effect of RBM3 silencing on cell cycle distribution was observed, as detected by flow cytometry following propidium iodide staining. **b** In T24 cells, RBM3 silencing resulted in an increase in G_1_-phase and a significant decrease in S-phase. **c** Relative mRNA levels of cell cycle checkpoint markers in RT4 and T24 cells transfected with siRNA targeting RBM3 compared to non-targeting siRNA control (dashed line). Mean ± SEM from at least four independent experiments, *p < 0.05, **p < 0.01, ***p < 0.001, two-tailed unpaired *t* test. **d** A schematic overview of the potential role of RBM3 in cell cycle progression based on findings from RNA-sequencing, cell cycle analysis as well as measurement of mRNA levels of selected cell cycle markers (red outline) after siRBM3 transfection in T24 cells

Following the results from the RNA-sequencing indicating a position of RBM3 in cell cycle regulation, particularly in proceeding from G_1_ to S-phase, cell cycle analysis by quantification of DNA content using flow cytometry was applied for further validation (Additional file 6: Fig. S4; Fig. 7a, b). In RT4 cells, RBM3 silencing had no effect on cell cycle distribution. Contrastingly, in T24 cells, a trend towards an accumulation of cells residing in G_1_-phase (*p* = 0.119) and a corresponding significant decrease in the percentage of cells in S-phase (*p* = 0.026) was demonstrated after siRBM3 transfection. G_1_-progression is sequentially orchestrated by several cyclins and cyclin-dependent kinases (CDKs), including activation of cyclin D (1, 2 and 3)/CDK4/6 in mid-G_1,_ and cyclin E/CDK2 in late G_1_. The catalytic activity of these cyclin/CDK complexes is under regulation of CDK inhibitors of the Ink4 and Cip/Kip family, such as p16, p18, and p27 [36]. The RNA-sequencing analysis of T24 cells indicated a linkage between RBM3 and cell cycle checkpoint markers associated with G_1_/S-phase transition, which was confirmed by qRT-PCR (Fig. 7c). In both cell lines, a significant upregulation of *CCND3* was seen upon siRBM3 treatment compared to control, whereas *CDK4* levels were significantly decreased. Additionally, in T24 siRBM3 cells, reduced levels of *CCND1* and *CDK2* and increased levels of *CDKN1B* were observed compared to control, thus confirming the results from the RNA-sequencing. Taken together, these findings suggest a functional role of RBM3 in facilitating cell cycle progression by promoting G_1_/S-transition (Fig. 7d).

## Discussion

In MIBC, interpatient heterogeneity in NAC response constitutes a formidable clinical issue, encouraging the search for accurate predictive biomarkers that could optimize treatment selection and hence, reduce the current overtreatment of non-responders. In the present study, we provide a first description of RBM3 as a potentially predictive biomarker of chemotherapy response in MIBC.

Previous studies have demonstrated upregulation of RBM3 in proliferating non-malignant cells [37] and in malignant compared to normal tissue, with high RBM3 protein expression also having been correlated with favourable prognosis in multiple solid malignancies [17]. Contrastingly, an inverse correlation has been denoted in pancreatic and other periampullary adenocarcinoma, where increased RBM3 protein levels were associated with more aggressive clinicopathological characteristics and a worse prognosis [18], as well as in hepatocellular carcinoma, where high RBM3 levels were shown to promote cellular proliferation, xenografted tumour growth, and signified an impaired survival [38]. In line with these findings, in the herein investigated cohort, patients with high tumour-specific RBM3 expression in TURB specimens had an inferior outcome with an increased risk of having recurrence of MIBC. However, and importantly, patients with high RBM3 expression who received NAC had a significantly reduced risk of recurrence and a prolonged survival compared to untreated patients. In addition, a trend towards a higher frequency of pathological downstaging after NAC treatment was observed in patients with high RBM3 expression compared to low expression, posing the question whether RBM3 might enhance chemosensitivity in MIBC. In support of this notion, RBM3 expression status has in several studies been recognized as a predictor of chemoresponsiveness, including gemcitabine and 5-fluorouracil (5-FU) in pancreatic adenocarcinoma [18], oxaliplatin-based chemotherapy in metastatic colorectal cancer [11] as well as of the effect of cisplatin in metastatic testicular non-seminomatous germ cell cancer [39] and epithelial ovarian cancer [16].

The indicated chemomodulating effects of RBM3 were further addressed using an in vitro model of two well-established human cancer cell lines commonly used in bladder cancer research. RT4 cells stem from a well-differentiated non-invasive papillary tumour [40], characterized as luminal [41] and p53 wildtype [42], whereas T24 cells are derived from a high-grade invasive transitional cell carcinoma [35], characterized by having mixed basal and luminal molecular features, and p53 mutation [42]. Both cell lines have previously displayed sensitivity to the herein used cytostatic agents [43]. In T24 urothelial cancer cells, which displayed markedly higher *RBM3* levels and a more efficient siRNA knockdown on the mRNA level than RT4 cells, siRBM3 transfection resulted in a significantly decreased sensitivity to both cisplatin and gemcitabine. These results are in line with previous studies on pancreatic cancer cells [18], in addition to ovarian cancer, where in vitro studies have shown significantly higher RBM3 levels in A2780 ovarian cancer cells compared to their cisplatin-resistant derivatives, and a decreased sensitivity to cisplatin after siRNA-mediated RBM3 suppression [16]. The cytotoxicity of cisplatin is primarily ascribed its interaction with purine bases of DNA to form crosslinks, resulting in activation of signal transduction pathways involved in DNA damage repair, cell cycle arrest and irreversible apoptotic programs [44]. In epithelial ovarian cancer, a functional description of RBM3 in the maintenance of DNA integrity, including regulation of DNA replication and chromatin remodeling, has been provided, suggesting that the indicated involvement of RBM3 in DNA damage may in part explain the correlation between RBM3 and sensitivity to cisplatin [45]. Moreover, the reduced cytotoxic effect of cisplatin in RBM3 silenced ovarian cancer cells has been reported to be mainly attributed to cell cycle alterations rather than to apoptosis, as cisplatin-induced G_2_/M-phase arrest was less evident in siRBM3-treated cells compared to control, whereas no significant changes in the percentage of apoptotic cells could be observed. This hypothesis was further supported by the lacking effect of RBM3 knockdown on the expression levels of the pro-apoptotic protein Bax [16]. Similarly, transcriptomic analysis of T24 cells revealed a functional association between RBM3 expression and cell cycle regulation, particularly at the G_1_/S-phase border. This was confirmed by flow cytometry where RBM3 silencing induced G_1_/S-arrest. RBM3 overexpression has previously been shown to relieve cell cycle arrest in the G_0_/G_1_-phase and cause cell transit into S-phase in neural stem cells [46]. RBM3 knockdown further resulted in altered levels of recognized regulators of G_1_-phase progression and/or G_1_/S-phase transition, including *CDK2, CDK4, CCND1, CCND3* and *CDKN1B,* which gives an insight into potential underlying mechanisms for the observed changes in the cell cycle distribution. In addition, a correlation between RBM3 and initiation of DNA replication was seen, possibly through interaction with the mini-chromosome maintenance (MCM)-complex which serves as a replicative helicase [47]. Thus, while further in-depth research is needed, these findings might provide some clues to the mechanistic basis through which RBM3 sensitizes cells to chemotherapy in urothelial carcinoma.

The prognostic implications of RBM3 in urothelial bladder cancer have been evaluated in a few earlier studies of mixed non-muscle-invasive and muscle-invasive tumours. Boman et al. have reported high RBM3 expression to be correlated with clinically less aggressive tumour characteristics and as an independent marker of improved survival [13, 14]. In a study by Florianova et al. [48], the relationship between RBM3 expression and less advanced tumour stages was confirmed, however, no prognostic significance could be observed. This discrepancy may be due to the use of different IHC assessment strategies, limited follow-up data as well as the larger proportion of advanced tumours in the latter study, as the prognostic value of RBM3 was found to be more evident in non-muscle-invasive tumours [13, 14]. In contrast to the present study, treatment status has not been previously accounted for, thus providing a possible explanation for the paradoxical relationship between upregulation of the proto-oncogene RBM3 and an improved outcome in advanced tumours. The herein presented results accordingly add to the accumulating notion of RBM3 as a predictive biomarker of chemotherapy response, also in MIBC. Yet, additional studies are warranted to elucidate whether high RBM3 expression is associated with a general sensitivity to chemotherapy or is restricted to the herein tested drugs. As for MIBC, the indicated predictive value of RBM3 in relation to gemcitabine merits particular attention. Apart from being recommended in combination with cisplatin in the neoadjuvant setting, gemcitabine can be administered to cisplatin-ineligible patients as first-line treatment [49]. Hence, patients with high tumour-specific RBM3 expression who are considered unfit for cisplatin-based chemotherapy may have a benefit from gemcitabine.

Furthermore, analysis of paired tissue samples yielded a higher RBM3 expression in TURB specimens compared to cystectomy specimens. Of note, a shift from low RBM3 levels at time of diagnosis to high RBM3 levels at time of radical cystectomy was shown to be restricted to a limited number of cases, indicating that evaluation of RBM3 expression in TURB specimens is a suitable method in terms of clinical decision-making. Herein, RBM3 expression was analysed using IHC, which offers several advantages over PCR or sequencing in the clinical setting as it is an easier, faster and less costly method. Moreover, IHC allows for biomarker analysis in a subcellular context, which is particularly relevant as it appears to be mainly the nuclear expression of RBM3 that carries prognostic significance in bladder cancer [13, 14]. *RBM3* mRNA levels were not assessed, however, comparison of RBM3 mRNA and protein levels has previously shown concordance [16, 18]. Further studies are needed to investigate if this also applies for RBM3 expression in MIBC.

The results from the present study stem from a consecutive well-annotated cohort of patients. In regard to the distribution of pathological/clinical T-stages at diagnosis and the administered chemotherapy, i.e. all NAC-treated patients having received MVAC regimen, it represents a homogenous study cohort which may explain why the response rates to NAC treatment are in the higher interval of previously reported data [20, 25, 50]. In terms of limitations, these are in line with the known limitations of a retrospective and experimental design. We acknowledge the potential influence of other factors on the prognostic benefit observed in NAC-treated patients with high tumoural RBM3 expression compared to untreated patients. As NAC-untreated patients were older [29] and considered ineligible for chemotherapy, they could have a worse prognosis regardless. As performance status was rarely registered in the medical records, no definitive conclusions can be made, and this should thus be denoted in subsequent studies. However, the included patients were considered fit for extensive surgical treatment and the prognostic analyses were based on TTR and CSS in addition to OS, limiting the influence of comorbidity on mortality. Future larger studies are needed to validate the results from this study, preferably also in relation to previously reported gene signature profiles and molecular subtypes of MIBC that have been shown to carry predictive significance [21–25]. In addition, follow-up studies of the mechanistic effect of RBM3 in MIBC could provide further valuable insights into its suggested role in chemosensitivity, e.g. by using additional cell lines representative of muscle-invasive disease and exploration of downstream pathways next to cell cycle progression.

## Conclusions

The herein presented clinical data provide a clear indication of the beneficial effect of neoadjuvant chemotherapy in urothelial tumours expressing high nuclear RBM3 levels. An observation further corroborated by in vitro experiments where siRBM3 transfected T24 high-grade urothelial cancer cells displayed a reduced sensitivity to both cisplatin and gemcitabine.

Transcriptomic analysis revealed a functional description of RBM3 in facilitating cell cycle progression by promoting G_1_/S-phase transition in these cells. Future investigations are of significant interest in order to further characterize the mechanisms of action of RBM3 as well as to delineate the potential clinical utility of RBM3 as a predictive biomarker of chemotherapy response in MIBC.

## Supplementary Information

**Additional file 1**: **Figure S1**. RBM3 protein expression in paired tissue specimens of MIBC. Spaghetti plots of the distribution of nuclear RBM3 expression in paired tissue specimens in a) the entire cohort and b) stratified according to NAC treatment. *P*-values were calculated using Wilcoxon signed-rank test, significant *p*-values are highlighted in bold. TURB, transurethral resection of the bladder.

**Additional file 2**: **Table S1.** Associations between RBM3 expression and clinicopathological characteristics in the entire cohort.

**Additional file 3**: **Table S2.** Sub-analysis of the associations between RBM3 expression and clinicopathological characteristics in strata according to TURB-only and paired tissue specimens.

**Additional file 4**: **Figure S2.** Original Western blots used for Fig. 3d. Three sets of samples from independent experiments were run in parallel separated by ladders for a) RT4 and b) T24 cells, respectively. Left images show total protein content with Revert total protein stain. Red line indicates cut prior to antibody incubation and detection. Right images display protein expression for loading control and protein of interest after detection. The red boxes indicate the cropped regions used in Fig. 3d.

**Additional file 5**: **Figure S3**. Comparison of cell viability after siRBM3 transfection. Cell viability of siRBM3 transfected a) RT4 and b) T24 bladder cancer cells compared to control (non-targeting siRNA) at 24, 30 and 72 h after transfection measured by WST-1 assay. No significant differences in cell viability were observed after siRBM3 transfection. Data represent mean ± SEM from at least three independent experiments performed in triplicate.

**Additional file 6**: **Figure S4**. Cell cycle analysis of RT4 and T24 bladder cancer cells. a) Representative flow cytometry scatter plots visualizing the gating strategy for cell population identification and doublet discrimination. Following transfection of RT4 and T24 cells with siRBM3 or non-targeting control, data were collected for 2 × 10^4^ cells for each sample, the cell population was gated and doublet discrimination was performed to identify single cells. b) The Watson Pragmatic algorithm was applied for identification of G1, S and G2/M cell populations.

## Data Availability

RNA-sequencing data are deposited at the NCBI Gene Expression Omnibus database [https://www.ncbi.nlm.nih.gov/geo/] under the accession number GSE167558. Access to all other data generated or analysed during this current study will be evaluated according to Swedish legislation and be made available from the corresponding author on reasonable request.

## Abbreviations

CI: Confidence interval
DEGs: Differentially expressed genes
GO: Gene ontology
HR: Hazard ratio
IHC: Immunohistochemistry
MCM: Mini-chromosome maintenance protein complex
MIBC: Muscle-invasive bladder cancer
NAC: Neoadjuvant chemotherapy
RBM3: RNA-binding motif protein 3
TMA: Tissue microarray
TTR: Time to recurrence
TURB: Transurethral resection of the bladder
