# Supplementary material for: Pre-clinical and clinical studies on the role of RBM3 in muscle-invasive bladder cancer: longitudinal expression, transcriptome-level effects and modulation of chemosensitivity
Source: BMC Cancer. 2022 Feb 2;22:131. doi: 10.1186/s12885-021-09168-7 (PMC8811987; doi:10.1186/s12885-021-09168-7)
Supplement: Supplementary file 1 — Additional file 1: Figure S1. RBM3 protein expression in paired tissue specimens of MIBC. Spaghetti plots of the distribution of nuclear RBM3 expression in paired tissue specimens in a) the entire cohort and b) stratified according to NAC treatment. P-values were calculated using Wilcoxon signed-rank test, significant p-values are highlighted in bold. TURB, transurethral resection of the bladder. [file 12885_2021_9168_MOESM1_ESM.pdf]

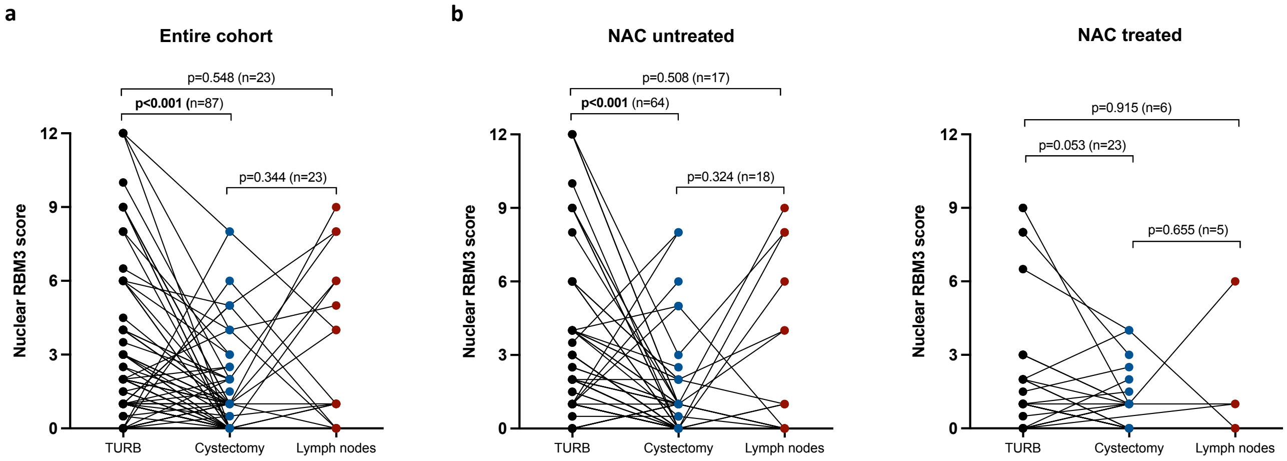

Figure S1. RBM3 protein expression in paired tissue specimens of MIBC. Spaghetti plots of the distribution of nuclear RBM3 expression in paired tissue specimens in a) the entire study cohort and b) stratified according to NAC treatment. *P*-values were calculated using Wilcoxon signed-rank test, significant *p*-values are highlighted in bold. TURB, transurethral resection of the bladder.
