# Supplementary material for: Pre-clinical and clinical studies on the role of RBM3 in muscle-invasive bladder cancer: longitudinal expression, transcriptome-level effects and modulation of chemosensitivity
Source: BMC Cancer. 2022 Feb 2;22:131. doi: 10.1186/s12885-021-09168-7 (PMC8811987; doi:10.1186/s12885-021-09168-7)
Supplement: Supplementary file 2 — Additional file 2: Table S1. Associations between RBM3 expression and clinicopathological characteristics in the entire cohort. [file 12885_2021_9168_MOESM2_ESM.pdf]

Table S1. Associations between RBM3 expression and clinicopathological characteristics in the entire cohort

	TURB			Cystectomy		
**RBM3 expression**	Low	High	*p-value adjusted*	Low	High	*p-value adjusted*
n of patients(%)	84(59.6)	57(40.4)		69(77.5)	20(22.5)	
**Age at diagnosis**						
Median (IQR)	69.7(13.6)	70.8(10.2)	*1.000*	72.8(8.4)	73.0(10.7)	*1.000*
Range	43.1-82.6	38.7-82.7		38.7-82.7	53.2-81.5	
**Sex**						
Female	14(16.7)	16(28.1)	*1.000*	15(21.7)	6(30.0)	*1.000*
Male	70(83.3)	41(71.9)		54(78.3)	14(70.0)	
**Pathological T-stage TURB specimens**						
pT1	3(3.6)	3(5.3)	*1.000*	4(5.8)	1(5.0)	*1.000*
pT2	70(83.3)	49(86.0)		57(82.6)	16(80.0)	
pT3	8(9.5)	2(3.5)		5(7.2)	2(10.0)	
pT4	3(3.6)	3(5.3)		3(4.3)	1(5.0)	
**Clinical T-stage TURB specimens**						
T2	38(45.2)	36(63.2)	*1.000*	29(42.0)	13(65.0)	*0.690*
T3	33(39.3)	16(28.1)		28(40.6)	7(35.0)	
T4	13(15.5)	5(8.8)		12(17.4)	0(0.0)	
**Pathological T-stage cystectomy specimens**						
pT0/pTa/CIS only	22(26.2)	20(35.1)	*1.000*	4(5.8)[a]	1(5.0)[a]	*0.891*
pT1	10(11.9)	3(5.3)		5(7.2)	6(30.0)	
pT2	15(17.9)	14(24.6)		14(20.3)	4(20.0)	
pT3	27(32.1)	14(24.6)		33(47.8)	7(35.0)	
pT4	10(11.9)	6(10.5)		13(18.8)	2(10.0)	
**N-stage**						
N0	59(70.2)	42(73.7)	*1.000*	46(66.7)	10(50.0)	*1.000*
N1	11(13.1)	5(8.8)		8(11.6)	4(20.0)	
N2	5(6.0)	6(10.5)		6(8.7)	3(15.0)	
N3	9(10.7)	4(7.0)		9(13.0)	3(15.0)	
**M-stage**						
0	74(97.4)	55(98.2)	*1.000*	62(98.4)	18(94.7)	*1.000*
1	2(2.6)	1(1.8)		1(1.6)	1(5.3)	
Missing	8	1		6	1	
**LVI in cystectomy specimens**						
Not present	57(91.9)	35(97.2)	*1.000*	59(89.4)	17(100.0)	*1.000*
Present	5(8.1)	1(2.8)		7(10.6)	0(0.0)	
Missing	22	21		3	3	
**CIS in TURB specimens**						
Not present	76(90.5)	50(87.7)	*1.000*	64(92.8)	15(75.0)	*0.297*
Present	8(9.5)	7(12.3)		5(7.2)	5(25.0)	
**CIS in cystectomy specimens**						
Not present	69(82.1)	48(84.2)	*1.000*	52(75.4)	18(90.0)	*1.000*
Present	15(17.9)	9(15.8)		17(24.6)	2(10.0)	
**Neoadjuvant chemotherapy**						
Yes	39(46.4)	26(45.6)	*1.000*	17(24.6)	6(30.0)	*1.000*
No	45(53.6)	31(54.5)		52(75.4)	14(70.0)	

[a]Cystectomy specimens with pT0 were excluded from the analysis (n=35). CIS, carcinoma in situ. LVI, lymphovascular invasion. Holm-Bonferroni adjusted *p*-values, significance level <0.05. The percentages refer to the distribution of clinicopathological characteristics within each category (low/high RBM3 expression).
