# Supplementary material for: Pre-clinical and clinical studies on the role of RBM3 in muscle-invasive bladder cancer: longitudinal expression, transcriptome-level effects and modulation of chemosensitivity
Source: BMC Cancer. 2022 Feb 2;22:131. doi: 10.1186/s12885-021-09168-7 (PMC8811987; doi:10.1186/s12885-021-09168-7)
Supplement: Supplementary file 3 — Additional file 3: Table S2. Sub-analysis of the associations between RBM3 expression and clinicopathological characteristics in strata according to TURB-only and paired tissue specimens. [file 12885_2021_9168_MOESM3_ESM.pdf]

Table S2. Sub-analysis of the associations between RBM3 expression and clinicopathological characteristics in strata according to TURB-only and paired tissue specimens.

	TURB-only			Paired TURB-cystectomy					
	TURB			TURB			Cystectomy		
**RBM3 expression**	Low	High	p_{adj}	Low	High	p_{adj}	Low	High	p_{adj}
n of patients (%)	29(53.7)	25(46.3)		55(63.2)	32(36.8)		67(77.0)	20(23.0)	
**Age at diagnosis**									
Median (IQR)	65.6(8.8)	67.0(11.1)	*1.000*	72.8(11.6)	73.2(6.4)	*1.000*	72.8(8.2)	73.0(10.1)	*1.000*
Range	43.1-82.6	45.6-77.6		50.3-81.7	38.7-82.7		38.7-82.7	53.2-81.5	
**Sex**									
Female	4(13.8)	5(20.0)	*1.000*	10(18.2)	11(34.4)	*0.891*	15(22.4)	6(30.0)	*1.000*
Male	25(86.2)	20(80.0)		45(81.8)	21(65.6)		52(77.6)	14(70.0)	
**Pathological T-stage TURB specimens**									
pT1	0(0.0)	2(8.0)	*1.000*	3(5.5)	1(3.1)	*1.000*	3(4.5)	1(5.0)	*1.000*
pT2	26(89.7)	21(84.0)		44(80.0)	28(90.6)		56(83.6)	16(80.0)	
pT3	2(6.9)	1(4.0)		6(10.9)	1(3.1)		5(7.5)	2(10.0)	
pT4	1(4.3)	1(4.0)		2(3.6)	2(6.3)		3(4.5)	1(5.0)	
**Clinical T-stage TURB specimens**									
cT2	16(55.2)	17(68.0)	*1.000*	22(40.0)	19(59.4)	*1.000*	28(41.8)	13(65.0)	*0.530*
cT3	9(31.0)	6(24.0)		24(43.6)	10(31.3)		27(40.3)	7(35.0)	
cT4	4(13.8)	2(8.0)		9(16.4)	3(9.4)		12(17.9)	0(0.0)	
**Pathological T-stage cystectomy specimens**									
pT0/pTa/CIS only	20(69.0)	17(68.0)	*1.000*	2(3.6)	3(9.4)	*1.000*	4(6.0)[a]	1(5.0)[a]	*1.000*
pT1	1(3.4)	1(4.0)		9(16.4)	2(6.3)		5(7.5)	6(30.0)	
pT2	5(17.2)	6(24.0)		10(18.2)	8(25.0)		14(20.9)	4(20.0)	
pT3	2(6.9)	1(4.0)		25(45.5)	13(40.6)		31(46.3)	7(35.0)	
pT4	1(3.4)	0		9(16.4)	6(18.8)		13(19.4)	2(10.0)	
**N-stage**									
N0	26(89.7)	20(80.0)	*0.880*	33(60.0)	22(68.8)	*1.000*	45(67.2)	10(50.0)	*1.000*
N1	3(10.3)	1(4.0)		8(14.5)	4(12.5)		8(11.9)	4(20.0)	
N2	0	3(12.0)		5(9.1)	3(9.4)		5(7.5)	3(15.0)	
N3	0	1(4.0)		9(16.4)	3(9.4)		9(13.4)	3(15.0)	
**M-stage**									
0	25(86.2)	25(100.0)	*1.000*	49(98.0)	30(96.8)	*1.000*	61(98.4)	18(94.7)	*1.000*
1	1(3.4)	0		1(2.0)	1(3.2)		1(1.6)	1(5.3)	
Missing	3	0		5	1		5	1	
**LVI in cystectomy specimens**									
Not present	10(100.0)	7(100.0)	-	47(90.4)	28(96.6)	*1.000*	58(90.6)	17(85.0)	*1.000*
Present	0	0		5(9.6)	1(3.4)		6(9.4)	3(15.0)	
Missing	19	18		3	3		3		
**CIS in TURB specimens**									
Not present	27(93.1)	22(88.0)	*1.000*	49(89.1)	28(87.5)	*1.000*	62(92.5)	15(75.0)	*0.506*
Present	2(6.9)	3(12.0)		6(10.9)	4(12.5)		5(7.5)	5(25.0)	
**CIS in cystectomy specimens**									
Not present	25(86.2)	24(96.0)	*1.000*	44(80.0)	24(75.0)	*1.000*	50(74.6)	18(90.0	*1.000*
Present	4(13.8)	1(4.0)		11(20.0)	8(25.0)		17(25.4)	2(10.0)	
**Neoadjuvant chemotherapy**									
Yes	21(72.4)	21(16.0)	*1.000*	18(32.7)	5(15.6)	*0.891*	17(25.4)	6(30.0)	*1.000*
No	8(27.6)	4(84.0)		37(67.3)	27(84.4)		50(74.6)	14(70.0)	

[a]Cystectomy specimens with pT0 were excluded from the analysis (n=35). CIS, carcinoma in situ. LVI, lymphovascular invasion. Holm-Bonferroni adjusted *p*-values, significance level <0.05. "-", statistical analysis could not be performed. The percentages refer to the distribution of clinicopathological characteristics within each category (low/high RBM3 expression).
