# Supplementary material for: Pre-clinical and clinical studies on the role of RBM3 in muscle-invasive bladder cancer: longitudinal expression, transcriptome-level effects and modulation of chemosensitivity
Source: BMC Cancer. 2022 Feb 2;22:131. doi: 10.1186/s12885-021-09168-7 (PMC8811987; doi:10.1186/s12885-021-09168-7)
Supplement: Supplementary file 4 — Additional file 4: Figure S2. Original Western blots used for Fig. 3d. Three sets of samples from independent experiments were run in parallel separated by ladders for a) RT4 and b) T24 cells, respectively. Left images show total protein content with Revert total protein stain. Red line indicates cut prior to antibody incubation and detection. Right images display protein expression for loading control and protein of interest after detection. The red boxes indicate the cropped regions used in Fig. 3d. [file 12885_2021_9168_MOESM4_ESM.pdf]

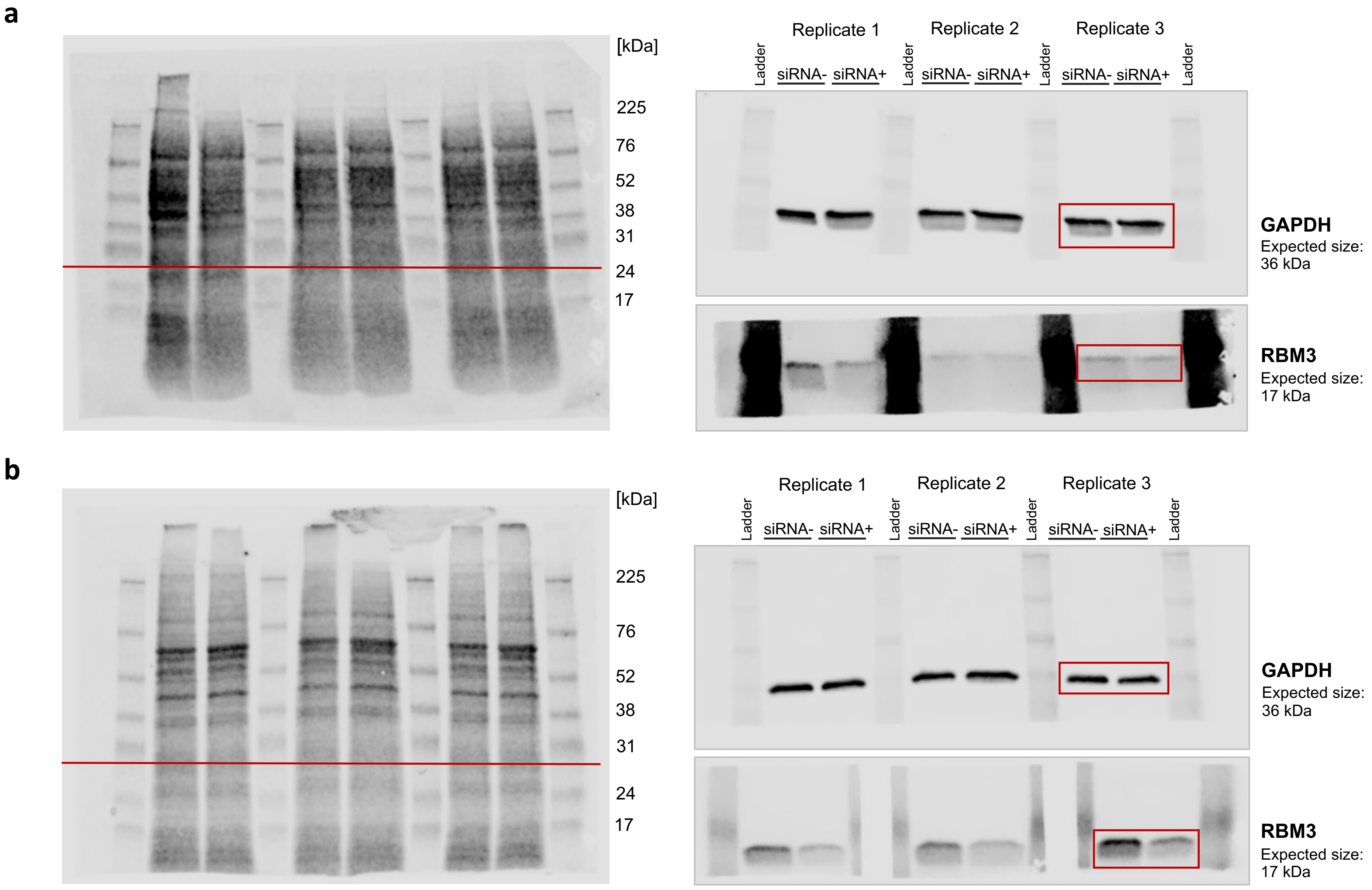

Figure S2. Original Western blots used for Figure 3d. Three sets of samples from independent experiments were run in parallel separated by ladders for a) RT4 and b) T24 cells, respectively. Left images show total protein content with Revert total protein stain. Red line indicates cut prior to antibody incubation and detection. Right images display protein expression for loading control and protein of interest after detection. The red boxes indicate the cropped regions used in Figure 3d.
