# Supplementary material for: Pre-clinical and clinical studies on the role of RBM3 in muscle-invasive bladder cancer: longitudinal expression, transcriptome-level effects and modulation of chemosensitivity
Source: BMC Cancer. 2022 Feb 2;22:131. doi: 10.1186/s12885-021-09168-7 (PMC8811987; doi:10.1186/s12885-021-09168-7)
Supplement: Supplementary file 5 — Additional file 5: Figure S3. Comparison of cell viability after siRBM3 transfection. Cell viability of siRBM3 transfected a) RT4 and b) T24 bladder cancer cells compared to control (non-targeting siRNA) at 24, 30 and 72 h after transfection measured by WST-1 assay. No significant differences in cell viability were observed after siRBM3 transfection. Data represent mean ± SEM from at least three independent experiments performed in triplicate. [file 12885_2021_9168_MOESM5_ESM.pdf]

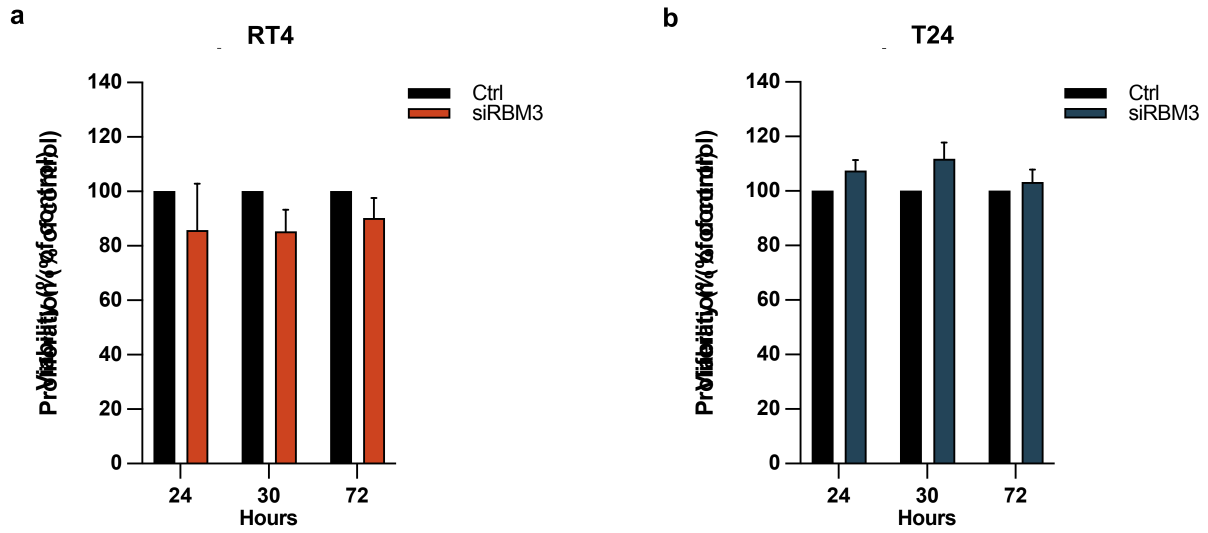

Figure S3. Comparison of cell viability after siRBM3 transfection. Cell viability of siRBM3 transfected a) RT4 and b) T24 bladder cancer cells compared to control (non-targeting siRNA) at 24, 30 and 72 h after transfection measured by WST-1 assay. No significant differences in cell viability were observed after siRBM3 transfection. Data represent mean ± SEM from at least three independent experiments performed in triplicate.
