# Supplementary material for: Pre-clinical and clinical studies on the role of RBM3 in muscle-invasive bladder cancer: longitudinal expression, transcriptome-level effects and modulation of chemosensitivity
Source: BMC Cancer. 2022 Feb 2;22:131. doi: 10.1186/s12885-021-09168-7 (PMC8811987; doi:10.1186/s12885-021-09168-7)
Supplement: Supplementary file 6 — Additional file 6: Figure S4. Cell cycle analysis of RT4 and T24 bladder cancer cells. a) Representative flow cytometry scatter plots visualizing the gating strategy for cell population identification and doublet discrimination. Following transfection of RT4 and T24 cells with siRBM3 or non-targeting control, data were collected for 2 × 10^4^ cells for each sample, the cell population was gated and doublet discrimination was performed to identify single cells. b) The Watson Pragmatic algorithm was applied for identification of G1, S and G2/M cell populations. [file 12885_2021_9168_MOESM6_ESM.pdf]

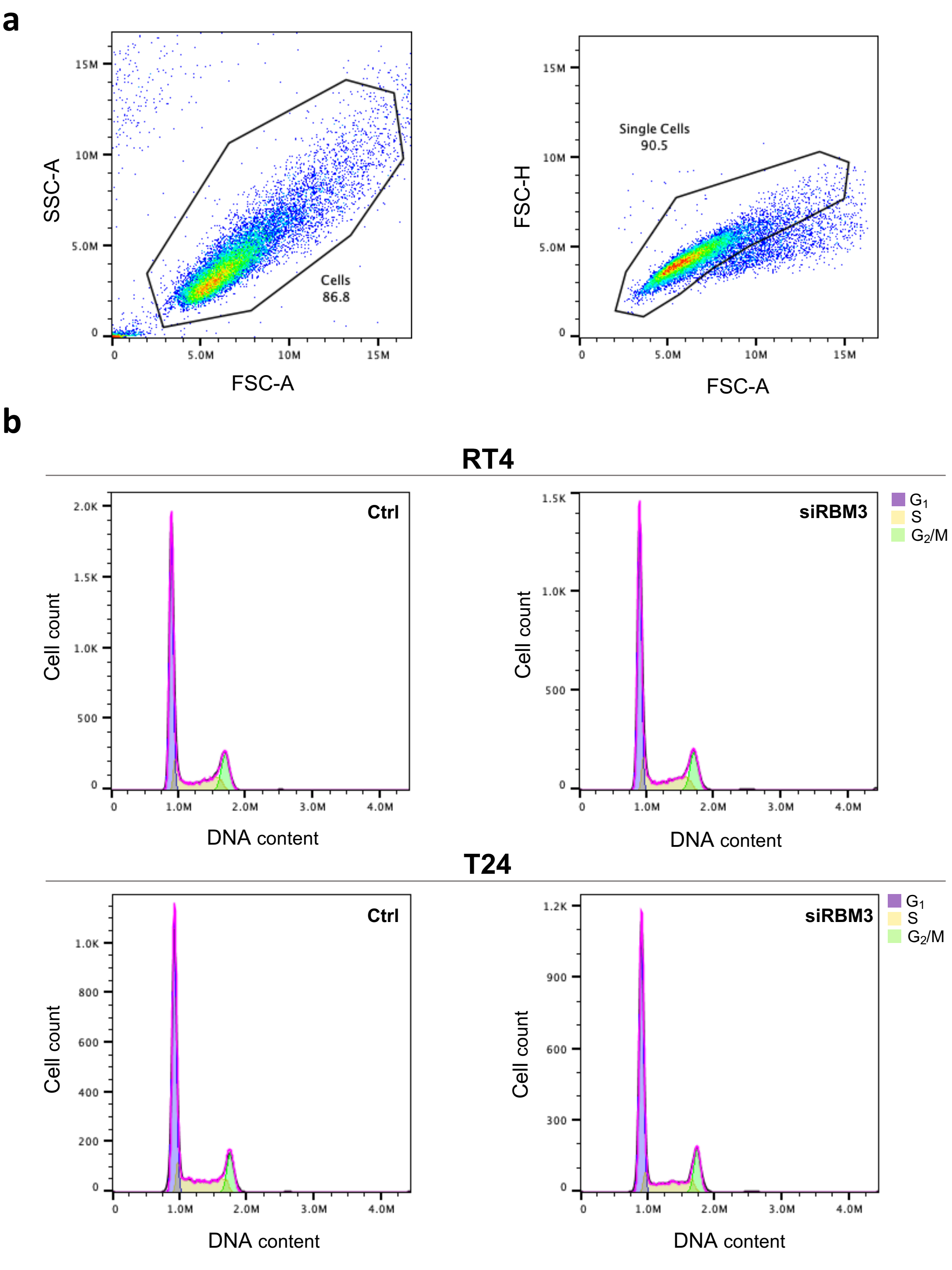

Figure S4. Cell cycle analysis of RT4 and T24 bladder cancer cells. a) Representative flow cytometry scatter plots visualizing the gating strategy for cell population identification and doublet discrimination. Following transfection of RT4 and T24 cells with non-targeting siRNA or siRBM3, data were collected for $2x10^4$ cells for each sample, the cell population was gated and doublet discrimination was performed to identify single cells. b) The Watson Pragmatic algorithm was applied for identification of G1, S and G2/M cell populations.
